# Using dried blood spot on HemoTypeSC™, a new frontier for newborn screening for sickle cell disease in Nigeria

**DOI:** 10.3389/fgene.2022.1013858

**Published:** 2022-10-26

**Authors:** Chinwe O. Okeke, Reuben I. Chianumba, Hezekiah Isa, Samuel Asala, Obiageli E. Nnodu

**Affiliations:** ^1^ Center of Excellence for Sickle Cell Research and Training, University of Abuja, Abuja, Nigeria; ^2^ Department of Medical Laboratory Science, Faculty of Health Sciences, University of Nigeria Nsukka, Nsukka, Enugu, Nigeria; ^3^ Department of Haematology and Blood Transfusion, College of Health Sciences, University of Abuja, Abuja, Nigeria; ^4^ Department of Anatomical Sciences, College of Health Sciences, University of Abuja, Abuja, Nigeria

**Keywords:** sickle cell disease, dry blood spot, newborn screening, HemoTypeSC, Hb Genotype, point-of-care-test (POCT)

## Abstract

**Background:** HemoTypeSC is a rapid, point-of-care testing (POCT) device for sickle cell disease (SCD) that traditionally uses the capillary blood from heel stick collected at the point of testing, a procedure that makes mass screening cumbersome and less cost-effective. Using dried blood spots (DBS) on HemoTypeSC could mitigate this challenge. Therefore, this study aimed to determine the feasibility of eluting blood from DBS to read on HemoTypeSC.

**Methods:** DBS and fresh samples from heel sticks were collected from 511 newborns at the immunization clinics of six Primary Health Centers in Abuja, Nigeria. The two samples from each newborn were analyzed using HemoType SC and then compared with the result of the isoelectric focusing (IEF) test.

**Results:** Of the 511 newborns, 241 were males and 270 were females. Standard HemoTypeSC (using fresh samples collected from heel sticks) and HemoTypeSC using DBS identified 404 (79.0%) HbAA, 100 (19.6%) HbAS, 6 (1.2%) HbSS, and 1 (0.2%) HbAC phenotypes. The IEF tests identified 370 (72.4%) HbAA, 133 (26.0%) HbAS, 5 (1.0%) HbSS, and 3 (0.6%) HbAC phenotypes. The sensitivity, specificity, positive predictive value (PPV), negative predictive value (NPV) and overall accuracy of HemoTypeSC using DBS, compared to standard HemoTypeSC POCT was 100%. IEF method showed for AA, AS, AC phenotypes; sensitivity; 84.7%, 67%,100% respectively, specificity; 67.6%, 86%, 99% respectively, PPV; 91.2%, 53%, 50% respectively, NPV; 52.7%, 91%, 100% respectively. For SS phenotype, IEF showed 100% specificity, sensitivity, PPV and NPV.

**Conclusion:** HemoTypeSC test using dried blood spot is as accurate as the standard point-of-care HemoTypeSC test. The use of DBS on HemoTypeSC could ensure better efficiency and cost-effectiveness in mass newborn screening for SCD.

## 1 Introduction

Sickle cell disease (SCD) is a genetic blood disorder with high prevalence in Sub-Saharan Africa (Nnodu et al., 2021). It is estimated that 3,12,000 newborns were born with sickle cell anemia globally in 2010, with 2,30,000 being born in Sub-Saharan Africa, accounting for 80 percent of the global sickle cell anaemia population (Berger et al., 2022) (Lanzkron, Patrick Carroll and Haywood, 2013). In high-income countries, the life expectancy of SCD patients has increased dramatically over the last 40 years, reaching 50 years. Whereas in Sub-Saharan Africa, most children with SCD are thought to die before reaching the age of five (Ware et al., 2017). Predictably between 2010 and 2050, the overall number of births affected by SCD will be 14,242,000. Specifically for Nigeria, the number is likely going to rise from 91,000 newborns with SCA in 2010 to 1,40,800 with SCA in 2050 (Piel et al., 2013). It is expected that large-scale universal screening stands the chance of saving up to 9,806,000 newborns with SCA globally, 85% of these newborns will be born in sub-Saharan Africa (Piel et al., 2013). SCD burden is high in Africa with especially high mortality amongst the under-fives. The prevalence of sickle cell trait in Nigeria is 25% and that of homozygous state is up to 2% in some regions. Nigeria is the country that has the highest burden of SCD (NDHS, 2018). Model estimates from the Nigeria National Demographic Survey showed that the national average under-5 mortality for children with SCD born between 2003 and 2013 was 490 per 1,000 livebirths (95% CI 270–700), 4·0 times higher (95% CI 2·1–6·0) than children with HbAA, with about 4·2% (95% CI 1·7–6·9) of national under-5 mortality attributable to excess mortality from SCD (Hsu et al., 2018) (Nnodu et al., 2021). In high-prevalence areas, there is evidence of several benefits of universal newborn screening (NBS) for SCD (Green et al., 2016).

Except for Egypt, many African nations lack a national NBS program. In the past, the Republics of Benin and Ghana were the only countries in Africa with SCD NBS programs, and even those are not at national levels despite the burden of SCD on the continent and the benefits of NBS with SCD management (Rahimy et al., 2009). Activities in the other countries include a variety of NBS pilot studies (Therrell et al., 2020).

Thus, there is a need for NBS programs to be scaled up nationally in most African countries. In Nigeria, the groundwork for a nationwide program has already been laid, but hampered by inadequate funding, high cost of reagents, and a lack of skilled manpower amongst other obstacles (Hsu et al., 2018). The fact that the bulk of the people in SSA live in rural regions and lacks access to healthcare is one of the most significant difficulties (National Population Commission (NPC) [Nigeria] and ICF, 2019). Point-of- care test (POCT) devices are reliable, easy-to-use and cheap, hence can considerably facilitate the identification of individuals with SCD in Nigeria and other countries in which the SCD prevalence is high (Nnodu et al., 2019).

A few POCT devices for SCD have recently been developed based on differential erythrocyte density (Kumar et al., 2014), differential mobility of Hb S and Hb A through filter paper (Yang et al., 2013), and a polyclonal antibody-based capture immunoassay (Kanter et al., 2015). All of these have one challenge or the other. Some of these challenges are either that the devices require apparatus as an inherent element of the technique to attain maximal specificity and sensitivity, or because of their lack of accuracy (Bond et al., 2017). A unique POCT (HemoTypeSC™ uses monoclonal antibodies (MAb) to distinguish between normal adult haemoglobin (HbA), sickle haemoglobin (HbS), and haemoglobin C (HbC) (Quinn et al., 2016). One of the first reports was in the evaluation of 100 whole blood samples from individuals with common relevant Hb phenotypes. HemoTypeSC was proven to be 100 percent accurate in identifying the proper Hb phenotype (Quinn et al., 2016). Since these antibodies are blind to haemoglobin F (HbF), they can reliably diagnose neonates with increased HbF but low levels of HbA or HbS.

In a study by Nnodu et al. (2019), the overall accuracy, specificity, and sensitivity of HemoTypeSC in identifying Hb phenotypes (AA, AS, AC, SS, SC, and CC) across multiple Nigerian primary healthcare centers in a real-life, field setting were evaluated. The results obtained in this study corroborated previously published findings and revealed a sensitivity and specificity of 100 percent for HbS and HbC, using high-performance liquid chromatography (HPLC) method as gold standard.

Dried blood spot (DBS) is a minimally invasive blood sampling technique. Blood samples are collected from the heel of newborns and applied into a cellulose or polymer card paper. The blood loaded card paper is air dried, after which it is stored in low gas-permeability plastic bags containing desiccant to reduce humidity. DBS is one of the most convenient tools for blood sample collection. Its benefits include; analytical measurements for more than 50 (Fifty) analytes, the sample has been found to be stable for a couple of months at ambient temperature or refrigeration with loss of enzymatic activity to a negligible extent, easy shipment zip-lock bags requiring no cold chain from sampling point to the laboratory and reduced risk of infection as a result of contaminated samples (Saud, 2018). Thus, this is an ideal sampling method in resource-poor settings. DBS sample has an economical preference for many clinical applications (Chace and Hannon, 2016). DBS has been used successfully on isoelectric focusing method (IEF) (Williams, 2016), and HPLC (Inusa et al., 2015).

HemoTypeSC is one of the POCT devices for SCD that has been extensively investigated and found with commendable performance characteristics. The normal HemoTypeSC procedure makes use of fresh capillary blood; hence screening has to be on the spot. Moreover, to reduce the turnaround time, two or three personnel have to be involved in the process, hence making it more cost implicative. These factors reduce the general effectiveness of the normal HemoTypeSC™ technique for use in a mass screening settings like immunization centers in resource limited countries.

Using fresh capillary blood sample for running HemoTypeSC technique may not provide the required efficiency needed in a mass screening setting. Considering the afore stated challenges, Dry blood sampling may be the way forward.

Here, we tried to determine the possibility of eluting blood from DBS to read HemoType SC™ compared to the standard method of using fresh capillary blood as applied in POCT.

## 2 Materials and methods

This is a pilot study and the aim is to find out the possibility of eluting blood from DBS to run HemoTypeSC™ protocol and to compare the results obtained with standard HemoTypeSC™ POCT and IEF method. Newborns zero (0) to six (6) weeks of age drawn across six immunization centers in the Federal capital territory (FCT) Abuja participated in the study.

### 2.1 The test methods

#### 2.1.1 HemoTypeSC™

Monoclonal antibodies (Mab) are used in the competitive lateral flow immunoassay known as HemoTypeSC™ to detect the presence of hemoglobin A, S, and C. The hemoglobin phenotypes HbAA, HbSS, HbSC, HbCC, HbAS, and HbAC are quickly detected using it (Bassimbié Kakou Danho et al., 2021). Each MAb bound just its target in a competitive enzyme-linked immunosorbent test with just 1.0% cross-reactivity. Since these antibodies are blind to haemoglobin F (HbF), it is possible to diagnose neonates with elevated HbF and low levels of HbA or HbS (Nnodu et al., 2019).

A cellulose wick, antibody-impregnated nitrocellulose, and laminated fiberglass sample pads make up test strips, which allow liquid samples to pass through the three components in a particular order. The process involved rehydrating the dried gold conjugate and dilution of the lysed blood sample using an assay solution that contained detergents and non-specific blocking reagents (Quinn et al., 2016). The presence of a line on the strips indicates the absence of the hemoglobin variant in the blood sample (Bassimbié Kakou Danho et al., 2021).

#### 2.1.2 Isoelectric focusing

IEF employs an agarose gel that enables qualitative and semi-quantitative analysis by separating various haemoglobins from a patient sample into distinct bands based on their isoelectric point. Haemoglobins are separated on one axis using IEF gel. Visual comparison of the individual bands to the closest reference samples is a typical method of qualitatively measuring patient sample.

#### 2.1.3 High-performance liquid chromatography

The principle of HPLC is based on the distribution of the analytes between a stationary phase such as the packing in a column and a mobile phase which is the sample or analytes which is pumped through a valve at high pressure. The interaction between the sample and the stationary phase or column depends on the chemical structure of the analyte which allow some molecules to be retained while some pass through more easily. The analyte is detected after leaving the column with the signals converted and recorded by a computer software in the form of a graph in wavelengths called a chromatograph. This method can be used to separate and quantitate haemoglobin and its variants. It is particularly sensitive to the detection Hb A2, Hb F.

Ethical clearance was obtained from Federal Capital Territory Research Ethics Committee. 511 newborns were tested at 6 immunization centers in the FCT.

The sampling was carried out between October 2021 and January 2022. Mothers of all eligible babies coming for immunization at the centers were approached for testing. Informed signed consent was obtained. The “Standard Precautions” protocol developed by the US Centers for Disease Control and Prevention was followed throughout the sample collection and testing to prevent infection when working with human blood samples (Quinn et al., 2016).

### 2.2 Storage, sampling and testing

The HemoTypeSC test kits containing the lateral flow assay (LFA) test strip, a transfer pipette, a sample cup, and a volumetric inoculation loop were stored at room temperature. HemoTypeSC is considered to be stable in high heat and does not require refrigeration.

Blood samples from babies 6 weeks and below were drawn by heel-prick into labeled filter paper cards unto a HemoTypeSC™ POCT Sample collection strip, supplied by Silver Lake Research Corporation. The POCT was carried out on site, while the blood spots were air dried for a minimum of 3 h at 18°C–25°C, shipped to the Centre of Excellence for Sickle Cell Disease Research and Training (CESRTA), University of Abuja Newborn Screening Laboratory and stored in gas-impermeable zipper bag, containing desiccant sachets and kept in the Refrigerator at −20°C. Iso electric focusing testing was performed at CESRTA lab using DBS. After 1 week, the dried blood sample was eluted and HemoTypeSC™ standard protocol followed to determine the test result. The tests were carried out strictly following the manufacturer’s instructions and test results were interpreted based on a reference chart provided by the manufacturer. Clinical control samples of previously-diagnosed AA, AS, SS, and SC individuals were included with each batch of HemoTypeSC and IEF to assess the performance of these techniques. Results from HemoTypeSC standard POCT, HemoTypeSC using DBS and IEF were then compiled in a spreadsheet for analysis.

### 2.3 Assessment

The sensitivity, specificity, positive and negative predictive values, and overall accuracy of HemoTypeSC using DBS was compared to standard HemoTypeSC POCT and IEF were calculated. Sensitivity was defined as 100% × TP/(FN + TP) specificity as 100% × TN/(FP + TN), positive predictive value as 100% × TP/(TP + FP), negative predictive value as 100% × TN/(TN + FN) and overall accuracy as (prevalence × sensitivity)/(1—prevalence) (specificity), where TP = number of true positive events, FP = number of false positive events, and TN = number of true negative events [18].

## 3 Results

A number of 241 males and 270 females were screened. The HemoTypeSC standard POCT protocol tests identified 404 HbAA (79.0%), 100 HbAS (19.6%), 6 HbSS (1.2%), and 1 HbAC (0.19%).

HemoTypeSC using DBS showed the same result pattern as that done using the standard POCT protocol. The test cannot differentiate Hb SS and sickle -β^0-^ thalassemia. No HbCC or HbSC were identified. Details per center and allele frequencies are presented in Table 1. The IEF tests identified 370 HbAA (72.4%), 133 HbAS (26.0%), 5 HbSS (0.97%), and 3 HbAC (0.58%). The results of the 84 discordant samples are displayed on Tables 2, 3 gives a summary of the frequency of the various haemoglobin phenotypes. Specificity, positive predictive value (PPV), negative predictive value (NPV) and overall accuracy of HemoTypeSC using dried blood spot, compared to standard HemoTypeSC were 100% as seen in Table 4. Isoelectric focusing (IEF) method showed; for AA, AS, AC Sensitivity; 84.7, 67, 100 respectively specificity; 67.6, 86, 99 respectively. Positive predictive value; 91.2, 53, 50 respectively. Negative predictive value; 52.7, 91, 100. For SS phenotype, IEF showed 100% specificity, sensitivity, positive predictive value and negative predictive value. Discordant results were found for a total of 84 samples. These 84 discordant samples were run with HPLC and the following results were obtained: 57 (AA), 21 (AS),1 (SS),1 (A3), 2 (ACS), 2 (DA). The discordant results were analysed by HPLC which showed that HemoTypeSC correctly identified all the HbAA, 21 of the HbAS but categorized 3 AD, 1 AC5, and 1 A3 as AS while IEF failed to identify 30 HbAA, wrongly labelling them as AS and was the only method to report 2 AC. Table 5 shows the measurement of agreement of Kappa between standard HemoTypeSC POCT and DBS HemoTypeSC and the measurement of agreement of Kappa between DBS HemoTypeSC and IEF. The table revealed Kappa value between HemoTypeSC POCT and DBS HemoTypeSC as 1.000 showing a strong agreement. The Kappa value between DBS HemoTypeSC and IEF was 0.540 showing a moderate agreement. For both measurements *p*-value <0.05 as seen in Table 5.

**TABLE 1:** Genotype and allele frequencies identified by HemoTypeSC and IEF in the 6 centers comprising University of Abuja Teaching Hospital immunization centers attached to it.

Id name *N*		Genotype											
		Standard POCT HemoTypeSC				DBS HemoTypeSC AA				1 EF			
		AA	AS	AC	SS	AA	AS	AC	SS	AA	AS	AC	SS
1 UATH	167	130 (77%)	35 (20%)	1	1	130	35	1	1	111	53	2	1
2 KHC	13	11 (84%)	2	—	—	11	2	—	—	12	1	—	—
3 TUNG	25	20	5	—	—	20	5	—	—	19	5	1	—
4 ORO	15	12	3	—	—	12	3	—	—	13	2	—	—
5 GTC	263	208	50	—	5	208	50	—	5	191	68	—	4

UATH, University of Abuja Teaching Hospital; KHC, Kutunku Health Center; TUNG, Tunga Maje Primary Health Center; ORO, Orozo Primary Health Center; GTC, Gwagwalada Town Clinic; AND, Angwuan Dodo Primary Health Center.

**TABLE 2:** Showing the results of the 84 discordant samples run with HPLC.

	HPLC	POCT HemoTypeSC	DBS HemoTypeSC	IEF
AA	57	57	57	27 (30)
AS	21	26	26	55 (30)
SS	1	1	1	—
AC	—	—	—	2
AD	3	—	—	—
AC5	1	—	—	—
A3	1	—	—	—
	84	84	84	84

**TABLE 3:** Showing the frequency distribution of the Hb phenotypes studied in the three methods used.

	(%)	(%)	IEF (%)
AA	404 (79.0)	404 (79.0)	370 (72.4)
AS	100 (19.6)	100 (19.6)	133 (26.0)
SS	6 (1.2)	6 (1.2)	5 (0.97)
AC	1 (0.19)	1 (0.19)	3 (0.58)

**TABLE 4:** Performance characteristics of DBS/POCT HemoTypeSC compared to IEF.

	Pheno- method TN type		FP	FN	TP	Sensitivity	Specificity	PPV	NPV
AA	STD HemoTypeSC/DBS	107	0	0	404	1.000	1.000	1.000	1.000
	HemoTypeSC								
	IEF	81	60	26	344	0.930	0.574	0.850	0.757
AS	STD HemoTypeSC/DBS	411	0	0	100	1.000	1.000	1.000	1.000
	HemoTypeSC								
	IEF	352	26	59	74	0.556	0.931	0.740	0.810
SS	STD HemoTypeSC/DBS	505	0	0	6	1.000	1.000	1.000	1.000
	HemoTypeSC								
	IEF	505	1	0	5	1.000	0.998	1.000	0.996
AC	STD HemoTypeSC/DBS	510	0	0	1	1.000	1.000	1.000	1.000
	HemoTypeSC								
	IEF	508	2	0	1	1.000	0.996	0.330	1.000

STD HemoTypeSC, Standard HemoTypeSC.

**TABLE 5:** Cohen’s Kappa Statistics showing the level of agreement of Kappa between DBS HemoTypeSC/Standard HemoTypeSC and DBS HemoTypeSC/IEF.

Symmetric measures						
			Value	Asymptotic standardized error^a^	Approximate T^b^	Approximate significance
Measurement of agreement	Kappa	Standard HemoTypeSC POCT/DBS HemoTypeSC	1.000	0.000	24.009	0.000
	—	DBS HemoTypeSC/IEF	0.540	0.042	13.184	0.000
No of valid ases			511			

a Asymptomatic standardized error.

b Approximate T.

Results revealed that most of the HPLC results of the discordant samples agree with the standard POCT and DBS HemoTypeSC results and not with the IEF results thus calling into question the validity of the designation of IEF/HPLC as gold standard methods (Nnodu et al., 2019, Nnodu et al., 2020).

## 4 Discussion

Our findings agree with previous studies suggesting a specificity and specificity of 100% (Nnodu et al., 2019) (Olatunya et al., 2021) for HbS and HbC in ideal conditions using the standard HemoTypeSC POCT procedure. Nnodu et al., worked on 1,121 samples and compared HemoTypeSC POCT with HPLC (GOLD Standard). They got a sensitivity of; 0.989 (AA), 0.983 (AS), 1.000 (SS), 0.933 (AC), and Specificity of: 0.993 (AA), 0.992 (AS), 0.999 (SS), 1.000 (AC). Olatunya et al. (2021) in 2021 compared Cellulose Acetate Electrophoresis, HemoTypeSC POCT and HPLC methods with PCR in 158 participants. HemoTypeSC showed both a Sensitivity and Specificity of 1.00 (100%) for AA, AC, AS, SC, and SS. Using DBS to run HemoTypeSC, our study produced a result that is 100% concordant with the standard HemoTypeSC procedure. This means that DBS samples can conveniently run on HemoTypeSC and accurate results produced. SSA countries like Nigeria, with high burden of SCD are faced with challenges of lack of accessibility to healthcare facilities for large portions of their populations. In such situations, primary care must become the focal point of SCD screening and treatment, with focus on initiatives that utilize user-friendly, reasonably priced technology and engage a sizable percentage of the community). No doubt certain attributes and qualities of POCT devices for sickle cell disease screening make it very suitable for use in SSA. These qualities are; kit not requiring rigorous procedure and expensive reagents, special skills and electricity not required.

The 84 Discordant results were run with HPLC which showed that HemoTypeSC correctly identified all the HbAA, 21 of the HbAS but categorised 3 AD, 1 AC5, and 1 A3 as AS. The results obtained by HPLC tally more with HemoTypeSC than with IEF as can be seen from Table 2; HPLC recorded 57 AA and HemoTpyeSC recorded 57 by both DBS and POCT methods, IEF recorded 27. HPLC recorded 21 AS with 5 variants; HemoTypeSC recorded 26 AS while IEF had 55 AS. From the results by the three methods, the ones by IEF seem as the outlier. Obviously using HPLC as gold standard, and referring to Table 3, IEF mis-identified 33 babies who had other phenotypes as having sickle cell trait.

There are still gaps in effective application of POCT in real life mass screening settings like immunization centers. As new knowledge is explored and meaningful collaborations developed, a situation arises when a battery of tests needs to be conducted as is applicable in the advanced countries, the use of these devices as POCT will not suffice, hence the application of DBS on such devices as HemoTypeSC will bridge these gaps.

### 4.1 Using dried blood spots on HemoTypeSC

Using DBS on HemoTypeSC has the potential of making the move of scaling up efforts to adopt early diagnosis, penicillin prophylaxis and hydroxyurea therapy, to forestall under5 mortality in SSA a reality. High level of discordance was discovered when DBS HemoTypeSC™ and IEF were compared on same subjects (16.4%). Among the 84 discordant samples, the AA and AS phenotype results by HPLC were in agreement with the standard HemoTypeSC POCT result. Only one positive AS sampled agreed with IEF. This goes to reveal the challenge associated with the high technicality involved with IEF and we infer that the IEF run in the Low- and middle-income countries may not be as efficient as those in the developed or high-income countries IEF method is the gold standard for newborn screening, the inconsistency observed in this study between IEF results and HPLC might be due to technical challenges facing the use of IEF in resource poor settings. Since DBS is utilized in other public health initiatives like HIV screening (Sikombe Id et al., 2019), it is possible that samples from early newborn diagnosis might be quickly and affordably tested for sickle hemoglobin using this technique. Sickle SCAN which is also is a rapid, qualitative, point-of-care lateral flow immunoassay for the identification of AS, AC, SS/Sβ^0^thal, SC, and CC/Cβ (Nguyen-Khoa et al., 2018). The limitation HemoTypeSC has is that, it does not detect or identify the β^0^ Thal phenotype.

The prevalence for SCD in this study HemoTypeSC both STD POCT and DBS was 1.2% and by IEF was 0.97%. This is within range of the reported prevalence of 1.4% and 1.2% from the same environment by Nnodu et al., 2020 and the 2018 NDHS. About 10 mothers declined their babies being screened for various reasons. Averagely, we can say that the screening apathy was due to lack of proper understanding of the importance of NBS. We suggest a more educational approach to tackle this problem. Other studies with similar sample sizes, came up with similar results, for instance; A study conducted in two selected primary health care centres in Shomolu local government area (LGA) in Lagos, Nigeria involving Two hundred and ninety-one mother-infant pairs presenting for the first or second immunization visit presented similar results. In this study, the haemoglobin genotype of mother-infant pairs was determined using the HemoTypeSC rapid test kit. Confirmation for the infants’ Hb genotype was carried out using HPLC. A SCD prevalence of the infant cohort was 0.8% not up to the proposed 2% for Nigeria (Oluwole et al., 2020). In a study conducted in Democratic Republic of Congo 448 children less than 5 years of age were screened. Among this number 12.7% were homozygous (SS) (Aimé et al., 2022). In the NDHS, there were areas of SCD prevalence up to 2% but that was not representative of the whole country.

## 5 Conclusion

HemoTypeSC test using dried blood spot is as accurate as the standard point-of-care HemoTypeSC test as can be seen from Table 5, with a Kappa value of 1.00. The use of DBS on HemoTypeSC could ensure better efficiency and cost-effectiveness in mass newborn screening for SCD. It can also provide an opportunity to leverage other public health programs such as the early infant diagnosis of HIV which utilize DBS to screen for SCD thus integrating the programs to expand SCD NBS services.

## Data Availability

The original contributions presented in the study are included in the article/supplementary materials, further inquiries can be directed to the corresponding authors.
